# Comparative Evaluation of the Antibacterial Efficacy of Type II Glass lonomer Cement, Type IX Glass lonomer Cement, and AMALGOMER™ Ceramic Reinforcement by Modified “Direct Contact Test”: An *in vitro* Study

**DOI:** 10.5005/jp-journals-10005-1345

**Published:** 2016-06-15

**Authors:** Shivayogi M Hugar, Harsha G Assudani, Vidyavathi Patil, Pratibha Kukreja, Chaitanya Uppin, Prachi Thakkar

**Affiliations:** 1 Professor, Department of Pedodontics and Preventive Dentistry KLE Vishwanath Katti Institute of Dental Sciences, Belagavi Karnataka, India; 2 Postgraduate Student, Department of Pedodontics and Preventive Dentistry KLE Vishwanath Katti Institute of Dental Sciences, Belagavi Karnataka, India; 3 Senior Lecturer, Department of Pedodontics and Preventive Dentistry KLE Vishwanath Katti Institute of Dental Sciences, Belagavi Karnataka, India; 4 Postgraduate Student, Department of Pedodontics and Preventive Dentistry KLE Vishwanath Katti Institute of Dental Sciences, Belagavi Karnataka, India; 5 Senior Lecturer, Department of Pedodontics and Preventive Dentistry KLE Vishwanath Katti Institute of Dental Sciences, Belagavi Karnataka, India; 6 Postgraduate Student, Department of Pedodontics and Preventive Dentistry KLE Vishwanath Katti Institute of Dental Sciences, Belagavi Karnataka, India

**Keywords:** AMALGOMER™ CR, Glass ionomer cement, Modified direct contact test, *Streptococcus mutans.*

## Abstract

**Background:** *Streptococcus mutans* (ATCC25175) has a profound effect on the incidence of dental decay in the human population. Many studies have been performed to assess the antimicrobial activity of different cements. However, little or no information is available about the antibacterial properties of Type II glass ionomer cement (GIC), Type IX GIC, and AMALGOMER™ ceramic reinforcement (CR).

**Aim:** To comparatively evaluate the antibacterial activity of Type II GIC, Type IX GIC, and AMALGOMER™ CR by modified direct contact test.

**Materials and methods:** The total sample size was 72 which was divided into four study groups. Six wells were coated by each: Type II GIC, Type IX GIC, AMALGOMER™ CR, and control group (only S. *mutans).* Statistical analysis was done using analysis of variance and the intergroup comparison was done using *post hoc* Tukey test.

**Results:** AMALGOMER™ CR was found to have a better antibacterial effect as compared with Type II and IX GIC.

**Conclusion:** AMALGOMER™ CR can serve as a valuable cement in pediatric dentistry due to its anticariogenic property.

**How to cite this article:** Hugar SM, Assudani HG, Patil V, Kukreja P, Uppin C, Thakkar P. Comparative Evaluation of the Antibacterial Efficacy of Type II Glass lonomer Cement, Type IX Glass Ionomer Cement, and AMALGOMER™ Ceramic Reinforcement by Modified “Direct Contact Test”: An *in vitro* Study. Int J Clin Pediatr Dent 2016;9(2):114-117.

## INTRODUCTION

Dental caries is a multifactorial local disease which involves destruction of the hard tissues of the teeth by metabolites produced by oral microorganisms. The uniqueness of dental caries makes it a fascinating study from a scientific standpoint.^1^

*Streptococcus mutans* has a profound effect on the incidence of dental decay in the human population. Under less severe sucrose exposure, the metabolic activity of *S. mutans* can potentiate the postprandial pH drop at the plaque-enamel interface, thereby interfering with the normal salivary remineralizing system and leading eventually to dental decay.

Several studies have been performed to assess the antimicrobial activity of different cements.^2,3^ However, little or no information is available about the comparison of antibacterial properties of Type II glass ionomer cement (GIC Type IX, GC Corporation, Tokyo, Japan, and AMALGOMER CR, Advanced Health Care, Tornbridge, United Kingdom.

The aim of the present study was to evaluate and compare the antibacterial efficacy of Type II GIC, Type IX GIC, AND AMALGOMER™ CR by modified direct contact test at 1-, 3-, and 7-day intervals.

## MATERIALS AND METHODS

This *in vitro* study was conducted in the Department of Pedodontics and Preventive Dentistry at KLE Vishwanath Katti Institute of Dental Sciences, Belagavi. Samples were processed in the KLE Dr Prabhakar Kore Basic Research laboratory of KLE University, Belagavi.

### Procedure

**Fig. 1:**
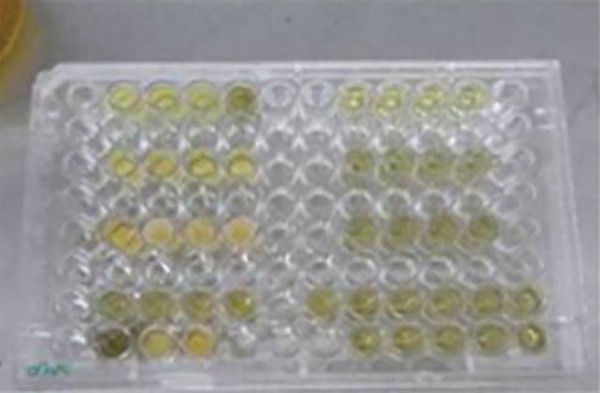
Microplate

The samples were processed by modified direct contact test on 96-well microplates (Fig. 1). Direct contact test is based on determining the turbidity of microbial growth in microplates.^4^ Facultative strains of *S. mutans* were grown on brain heart infusion (BHI) agar. Microorganisms were subcultured in appropriate culture media and under gaseous conditions to confirm their purity. Facultative strain was inoculated individually into tube containing 5 ml of sterile saline. The suspension was then adjusted to 0.5 McFarland scale = 1.5 × 10^8^ colony-forming units (CFU) spectrophotometrically at 630 nm. A 96-well microtiter plate was held vertically, and an area of fixed size on the wall of the six wells was coated with an equal amount of each material by using a cavity liner applicator. The materials were mixed in strict compliance with the manufacturers’ recommendations.

**Fig. 2:**
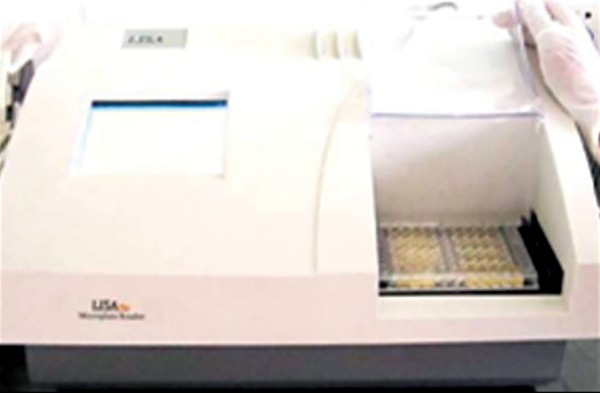
Microplate spectrophotometer

A 10 μl (approx 10^7^) bacterial suspension was placed in the coated wells (row A). After incubation for 1 hour in humidity at 37°C, the suspension liquid evaporated, ensuring direct contact between *S. mutans* and surface of tested material. Brain heart infusion broth (245 μl) was added to each of the wells and the plates were gently vortex-mixed for 2 minutes; 15 μl of bacterial suspension was then transferred from wells into an adjacent set of wells containing fresh medium (215 μl) and again mixed for 2 minutes. The kinetics of bacterial outgrowth in each well of rows A and B was measured at 630 nm using a microplate spectrophotometer (ELISA reader) (Fig. 2). Densitometric readings were taken hourly for 15 hours and with each set of samples.

Sample size for each material is 72 as the experiment is triplicated. Similar experimental procedures were carried out in which the tested material was allowed to age for 1st, 3rd, and 7th day in phosphate-buffered saline.

**Fig. 3:**
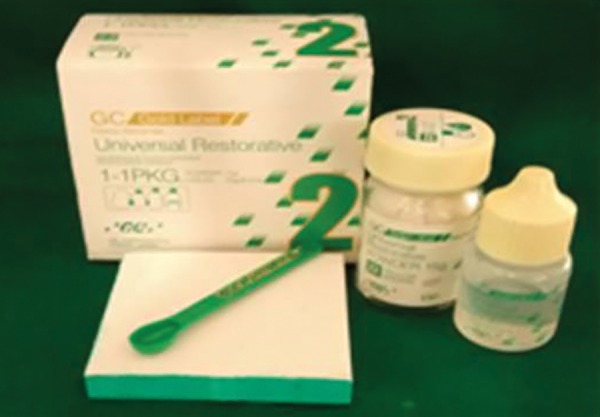
Type II glass ionomer cement

**Fig. 4:**
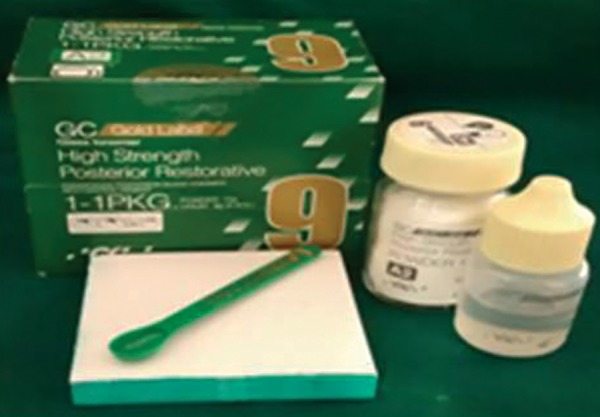
Type IX glass ionomer cement

### Study Groups

The samples were divided into four experimental groups:

Group 1 (control group): Six wells containing

*S. mutans* Group 2: Six wells coated with Type II GIC (Fig. 3)

Group 3: Six wells coated with Type IX GIC (Fig. 4)

**Fig. 5:**
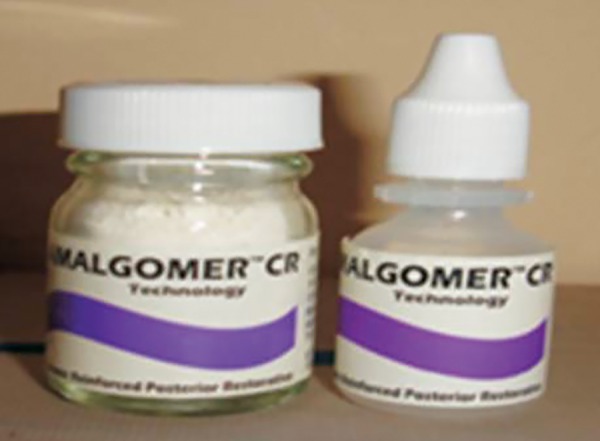
AMALGOMER™ CR

Group 4: Six wells coated with AMALGOMER™ CR Fig. 5).

## EXPERIMENTAL DESIGN

The wells were coated with freshly mixed tested material by using a cavity liner applicator. After 10 minutes, 10 μl bacterial suspension (10^8^ CFU) was placed on the test material. Wells were inspected for evaporation of the suspension’s liquid, which occurred within 1 hour at 37°C. Brain heart infusion agar (245 μl) was added to each of these wells and gently mixed for 2 minutes; 15 μl of broth was then transferred from subgroup 1 wells to an adjacent set of subgroup 2 wells that already contained fresh BHI medium (215 μl). Plate was placed for incubation at 37°C. Optical density readings were taken after 1 hour, 1st, 3rd, 5th, and 7th day in each well measured at 630 nm.

### Statistical Analysis

The data obtained were entered in Microsoft Excel sheet and all scores were calculated. Data analysis was done using Statistical Packages for the Social Sciences (SPSS) for windows 16.0 (SPSS Inc. Chicago, IL, USA). Analysis of variance with repeated measures was done to indicate differences between the experimental groups and the control group followed by *post hoc* Tukey test for intergroup comparison.

## RESULTS

In the present study, AMALGOMER™ CR showed the maximum amount of antibacterial activity followed by Type II GIC. Type IX GIC had the least antibacterial property against *S. mutans* as compared with the other two cements (Table 1). The results were statistically significant for AMALGOMER™ CR and Type II GIC.

## DISCUSSION

Secondary caries is a localized lesion occurring around restorations that is identical in etiology and histology to primary caries. Secondary caries process is difficult to diagnose and cannot be permanently treated by operative management. One method for reducing the frequency and severity of this issue is the use of fluoride-containing restorative materials.^5^ Glass ionomer cements have proven antibacterial activity against *S. mutans, S. oralis, S. salivarius,* and *Streptococcus* species. Clinical experience has indicated that very few or no secondary carious lesions are observed around the glass ionomer restorations.^6^

Gothenburg in June 2003 in his presentation at the International Association for Dental Research described AMALGOMER CR, Advanced Health Care, Tornbridge, United Kingdom complies with not only international standards for GIC, but with the standards for amalgam as well.^7^ It is manufactured by special process of improvization and treatment of the main glass ionomer components, fluoroaluminosilicophosphate glass and polyalkenoic acids.

The agar diffusion test (ADT) used to be the most commonly applied method to assess the antimicrobial activity of various cements. However, the limitations of this method are well recognized nowadays, and therefore, ADT is no longer a recommended method.^2,8,9^ A direct contact test which circumvents many of the problems of ADT was first introduced by Weiss et al. The test is quantitative and reproducible that allows testing of insoluble materials and can be used in standardized settings.^4^ The direct contact test may be a more suitable test than the ADT to evaluate antibacterial properties of definitive cements. Also, this test simulates the oral conditions unlike ADT. The method also allows for better control of possible confounding factors compared with ADT. It is essential to test the materials immediately after mixing and also after a period of time when it assumes its final chemical structure as release of various transitory and permanent products takes place. Both conventional and the resin-modified glass ionomers have been shown *in vitro* to reduce artificial caries and *in vivo^10^* to remineralize carious lesions^11^ and to enhance fluoride uptake by underlying dentin.^12^ The difference in antimicrobial patterns of various materials may depend upon the degree of setting. Hence, the antibacterial efficacy was evaluated at 1st, 3rd, and 7th day after mixing the cements.

**Table 1:** **Table 1:** Mean optical density of control group, Type II GIC, Type IX GIC, and AMALGOMER™ CR at 1st, 3rd, and 7th day

		*Control group (Group 1)*				*Type II GIC (Group 2)*				*Type IX GIC (Group 3)*				*AMALGOMER™ CR (Group 4)*			
		*Mean OD*		*SD*		*Mean OD*		*SD*		*Mean OD*		*SD*		*Mean OD*		*SD*	
Day 1		0.1068		0.0994		0.0924		0.0221		0.0971		0.0321		0.0811		0.0673	
Day 3		0.0813		0.0430		0.0923		0.0285		0.1070		0.0342		0.1058		0.0770	
Day 7		0.1539		0.0487		0.1240		0.0309		0.1601		0.0447		0.1210		0.0581	
p-value			0.993				0.030*				0.828				0.014*		

*Tukey test was applied for intergroup comparison

## CONCLUSION

All the test materials exhibited antibacterial activity against *S. mutans,* but to varying degrees. AMALGOMER™ CR was the most effective as compared with Type II and IX GIC at the end of 7 days. The antibacterial efficacy decreased over 3rd and 7th day. The antibacterial efficacy of AMALGOMER™ CR was the best followed by Type II and IX GIC.
